# Characterizing the Risk of Depression Following Mild Traumatic Brain Injury: A Meta-Analysis of the Literature Comparing Chronic mTBI to Non-mTBI Populations

**DOI:** 10.3389/fneur.2020.00350

**Published:** 2020-05-19

**Authors:** Sarah C. Hellewell, Caerwen S. Beaton, Thomas Welton, Stuart M. Grieve

**Affiliations:** ^1^Imaging and Phenotyping Laboratory, Charles Perkins Centre, Faculty of Medicine and Health, University of Sydney, Sydney, NSW, Australia; ^2^Sydney Medical School, University of Sydney, Sydney, NSW, Australia; ^3^Department of Radiology, Royal Prince Alfred Hospital, Sydney, NSW, Australia

**Keywords:** depression, concussion, mild traumatic brain injury, chronic mild traumatic brain injury, meta-analysis

## Abstract

**Objective:** Mild traumatic brain injury (mTBI) is associated with depressed mood acutely post-injury, but there is little evidence regarding long-term depression. The aim of this study was to determine the odds ratio (OR) of depression chronically following mTBI.

**Methods:** We searched Medline (PubMed), ProQuest, and Web of Science from date of database creation to January 23, 2019, for eligible studies examining depression at least 6 months post-injury in adult subjects with mTBI of any etiology, including civilians and military. Three authors independently reviewed titles and abstracts for study eligibility. Data were extracted and collated by two investigators. Risk of bias was assessed with the SIGN methodology. Study data were pooled using random-effects meta-analysis. The primary exposure was mTBI, and the primary outcome was depression. Secondary exploratory variables were time of assessment, age at injury, age at assessment, sex, and etiology.

**Results:** We included 47 cross-sectional studies (*n* = 25,103 mTBI and 29,982 control), 26 cohort studies (*n* = 70,119 mTBI, 262,034 control), four prospective observational studies (*n* = 1,058 mTBI and 733 control), two prospective longitudinal studies (*n* = 119 mTBI, 81 control), two case-control studies (*n* = 56 mTBI, 56 control), and one randomized controlled trial (*n* = 252 mTBI, 3,214 control). mTBI was associated with a 3.29-fold increased risk of depression (OR 3.29, 95% CI 2.68–4.03, *I*^2^ = 96%). The OR for depression did not change when subjects were assessed at 6–12 months (OR 2.43, 1.45–4.07), years 1–2 (OR 4.12, 2.10–8.07); 2–10 (OR 3.28, 2.42–4.46), or 10+ (OR 3.42, 1.51–7.77). Similar risk of depression was sustained across different age at injury (<25: OR 2.26, 1.82–2.81; 25–35: OR 4.67, 3.06–7.14; >35: OR 2.69, 1.42–5.10) and different age at assessment (<40 years: OR 3.14, 2.48–3.99; >40 years: OR 4.57, 2.54–8.24). Female sex had a non-significant increase in OR (OR 19.97, 2.39–166.93) compared to male (OR 3.0, 2.33–3.86). mTBI etiology had no impact on depression.

**Conclusions:** Those experiencing mTBI are more than three times more likely to experience depression compared to those without a history of mTBI, and this risk remains decades beyond the mTBI event. Future longitudinal studies are needed to identify and mitigate this risk.

## Introduction

Mild traumatic brain injury (mTBI) occurs at an incidence exceeding 42 million per year worldwide (1). mTBI is defined as “an acute brain injury resulting from mechanical injury to the head from external physical forces” (2). The causes of mTBI are numerous, including sporting collisions, blast neurotrauma, and motor vehicle accidents, among others (3). mTBI is associated with acute neuropsychiatric dysfunction, with depression a commonly described sequel. Many acute symptoms dissipate with resolution of the physiological injury; however, depression may persist (or develop) in the post-acute or chronic time frame (4). While much is known about the long-term physical and cognitive consequences of mTBI, the mechanisms underlying emotional dysregulation after mTBI are less understood (5).

Depression has many direct consequences, and elucidation of the burden attributable to depression is essential to understanding the impact of this disorder in the post-mTBI setting. Depression may mediate the relationships between mTBI and poor physical health (6), diminish functional capacity (7), impair executive control (4), and hinder social function (8). Depression also decreases quality of life after mTBI (7) and has a substantial personal and economic burden (9). In studies encompassing TBI of all severities, post-injury depression may occur in over 40% of subjects, with a relative risk of 7.5 (10, 11). The true prevalence of depression in the chronic period following mTBI is uncertain, with most studies focusing on mTBI in the acute phase (days–weeks) as part of a constellation of post-concussive symptoms or on moderate-to-severe injuries. Suggested risk factors for post-TBI depression are heterogeneous across available data; indeed, no clear consensus has emerged regarding such factors such as female sex (11–13) and age at injury (14, 15). Moreover, the likelihood of depression following mTBI sustained *via* different injury modalities has not been investigated.

Major depressive disorder (MDD) has a lifetime prevalence of 20.6% and known overlap with mTBI. Given that mTBI is an extremely common injury with an approximate lifetime prevalence of 22% [range: 12–46% (16)], the degree of association between mTBI and depression is therefore important to quantify. Evaluation of any causal link between mTBI and depression is problematic due to the heterogeneity of primary injuries and outcomes, late emergence of symptomology, and lack of formal large-scale trials in the area. In lieu of definitive large-scale longitudinal clinical assessments, a meta-analysis of literature spanning decades post-mTBI is the only realistic means to elucidate the association between mTBI and depression and identify the true odds of occurrence.

The aim of this study was to conduct a meta-analysis on all published data assessing depression after adult mTBI from a mean of 6 months post-injury onward to determine the lifetime odds ratio (OR) of depression chronically following an mTBI event.

## Methods

This study was conducted according to the Meta-analysis Of Observational Studies in Epidemiology (MOOSE) Checklist for Meta-analyses of Observational Studies criteria (eTable 1).

### Search Strategy

We performed a random-effects meta-analysis. Operational definitions of mTBI and depression [obtained through Medical Subject Headings (MeSH) thesauruses and non-MeSH terms] were used to determine the search strategy. Our initial search in PubMed contained the keywords “mild traumatic brain injury,” “depression,” and “mood disorder” (see eMethods for full search strategy). Literature was searched from the date of database inception to January 23, 2019.

### Selection Criteria

Studies were chosen according to the “PICOS” principles, with studies eligible if meeting the following criteria:

1. Population: Human adults assessed more than 6 months post-injury
2. Indicator: At least one instance of mTBI
3. Comparison: A control comparison group matched in age and sex
4. Outcome: Depression assessed using a measure validated in accordance with the standard operationalized diagnostic criteria [Feighner criteria, Research Diagnostic Criteria, Diagnostic, and Statistical Manual of Mental Disorders (DSM)-III, DSM-III-R, DSM-IV, DSM-5, and International Classification of Diseases (ICD)-10].
5. Study design: Original reports of case-control, cohort, cross-sectional, and prospective observational studies. Where possible, randomized controlled trials were included, provided that baseline (pretreatment) assessments were reported. Due to the nature of mTBI research, the vast majority of studies were non-randomized, thus we included randomized and non-randomized study designs.

### Data Extraction

Three investigators (SH, CB, TW) independently selected the studies and screened articles for exclusion on the basis of title and abstract, followed by review of full manuscripts and Supplementary Material. Studies examining moderate or severe TBI, combining injury severities, or including depression when present before injury were excluded. Studies reporting continuous data categorizing subjects as positive or negative for depression symptoms were examined for agreement in questionnaire cutoff values.

Studies straddling exclusion criteria were discussed for agreement among at least two authors. Three investigators (SH, CB, and TW) assessed the risk of bias. Any discrepancies at this stage were resolved by discussion among all authors until consensus was reached.

### Outcomes and Sub-analyses

Our primary outcome of interest was presence or absence of depression at any time beyond 6 months post-mTBI. Planned secondary analyses examined the ORs of (1) depression with increasing chronicity post-mTBI from 6 months onward; (2) influence of age at injury (under 25, 25–35, over 35); (3) influence of age at assessment (under 40, over 40); (4) relationship of biological sex and odds of depression; (5) whether mTBIs of different etiologies confer a different risk of depression; (6) whether repeated injuries increase the odds of post-mTBI depression.

### Risk of Bias and Study Heterogeneity

To assess the risk of bias in included studies, we combined the SIGN Methodology Checklist 3 for cohort studies and Checklist 4 for case-control studies. Heterogeneity was quantified using the *I*^2^ test statistic, where an *I*^2^ of below 50% indicates no/low heterogeneity, 50–75% moderate heterogeneity, and above 75% indicates high heterogeneity. Due to expected variation in effect sizes arising from methodological differences and sampling bias inherent to the type of studies examined, heterogeneity was handled by use of a random-effects model. Funnel plots were produced to assess publication bias, with calculation of Failsafe-N (17). Subgroup analyses (defined under “outcomes”) were also planned to determine whether differences in effect sizes were due to variations in methodology (18).

### Data Analysis

The following information was extracted for each study: measure of depression used in assessment and score for depression (dichotomous data: number classed as depressed for mTBI or control and total number of participants in each group; continuous data: mean and standard deviation for mTBI and control groups). For subgroup analysis, we also extracted mean time since mTBI, mean current age (with age at injury calculated from these two variables if not reported), and etiology of injury classified as sports mTBI, motor vehicle accident (MVA), military injuries or “other/unclassified.” In the case of multiple studies reporting data from the same cohort, we included only the study for which the most complete data set was available. We estimated summary ORs for dichotomous data, and mean differences (MDs) for continuous outcomes (see eMethods for further information). To combine these data sets, MDs were converted to ORs using the formula of Chinn (19). We used The Cochrane Collaboration RevMan software (20) to collate data and produce forest plots, funnel plots, and figures for risk of bias. One-way ANOVAs and Student's *T*-tests were calculated to determine statistical differences between subgroups using GraphPad Prism version 7.00 for Mac OS X (GraphPad Software, La Jolla, CA). Unless specified, *p*-values are representative of significance at the meta-analysis level.

## Results

**Figure 1:**
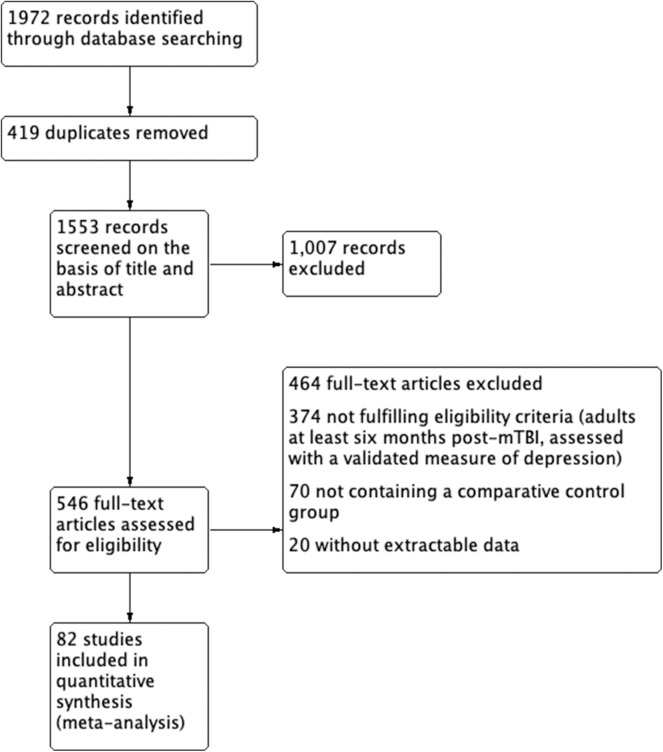
PRISMA flowchart of study selection process.

Figure 1 describes the screening process for 1,972 studies assessed for inclusion or exclusion in the meta-analysis. The 82 studies ultimately included in the meta-analysis comprised a total of 392,834 subjects. Thirty-seven studies reported assessment of post-mTBI depression using dichotomous data (361,814 subjects) while 45 reported findings as continuous data (30,991 subjects). Table 1 summarizes the characteristics of included studies. We included two case-control studies, one randomized trial, 48 cross-sectional studies, 26 cohort studies, and six prospective longitudinal studies. Of the total 392,834 subjects, 96,688 had mTBI and 296,117 were controls. Control subjects fell into the following categories: healthy control or population samples (28 studies), veterans or active military without mTBI (36 studies), sportsplayer peers or peers of cases (13 studies), and non-mTBI injuries or illnesses (five studies; see eResults 1 for further analysis). In the dichotomous data, the frequency of depression across the mTBI cohorts was 8% compared to 3% across the control cohorts.

**Table 1:** Characteristics of studies examining depression chronically after mTBI meeting the criteria for inclusion.

**Reference**	**Study type**	**Depression measure**	**Mean age at trauma**	**Mean age at follow-up (SD)**	**Mean time since trauma, years (SD)**	**% F**	**n mTBI**	**n control**	**Control type**
Amick et al. (21)	Cross-sectional	NSI-22	NS	34.0 (8.6)	NS	6	22,662	26,153	Veterans without mTBI Hx
Astafiev et al. (22)	Cross-sectional	CES-D	38.6	39.8 (11.8)	1.2 (1.5)	55	20	22	Healthy control
Baker et al. (23)	Case control	BDI-II	NS	56.7	NS	0	21	21	Active sportsplayer peers without mTBI Hx
Baldassrre et al. (24)	Prospective observational	BDI-II	27.2	32.0 (8.9)	4.8 (2.18)	11	188	210	Veterans without mTBI Hx
Barnes et al. (25)	Cross-sectional	BDI-II	NS	30.3 (8.2)	NS	0	46	46	Veterans without mTBI Hx
Bell et al. (26)	Cross-sectional	BDI	NS	38.7 (11.3)	NS	55	20	20	Migraine headache
Bomyea et al. (27)	Cross-sectional	BDI-II	NS	32.8 (6.8)	NS	14	52	32	Veterans without mTBI Hx
Bryant et al. (28)	Cohort	DSM-IV	37.8	38.8 (13.6)	1.0	28	321	321	Hospital sample - other trauma (non-mTBI)
Callahan et al. (29)	Cohort	PAS-DEP	NS	31.5 (7.6)	NS	2	42	36	Veterans without mTBI Hx
Carrier-Toutant et al. (30)	Cross-sectional	BDI-II	19.2	21.2 (2.49)	2.0 (0.7)	51	43	40	Active sportsplayer peers without mTBI Hx
Chi et al. (31)	Cohort	ICD-9	NS	NS	NS	42	55,456	221,501	Healthy control
Chong et al. (32)	Cohort	BDI	29.0	36.0	7.0	39	33	33	Healthy control
Coughlin et al. (33)	Cross-sectional	HAMD	24.3	31.3 (6.1)	7.0 (6.4)	0	14	16	Healthy control
Dailey et al. (34)	Cross-sectional	BDI	21.0	21.9 (2.8)	0.9 (0.3)	73	15	14	Healthy control
de Almeida Lima et al. (35)	Cross-sectional	HADS	37.5	39.0 (2.9)	1.5	49	36	36	Case-control pairing (friends/family)
Dean and Sterr (36)	Cross-sectional	HADS	25.7	26.7 (1.9)	1.0	0	36	36	General public
Decq et al. (37)	Cross-sectional	PHQ-9	NS	52.0	NS	0	217	158	Retired sportsplayer peers without mTBI Hx
Didehbani et al. (38)	Cross-sectional	BDI-II	NS	58.6 (10.3)	NS	0	30	29	Healthy control
Dismuke-Greer et al. (39)	Cohort	PHQ-9	28.7	38.0^*^	9.3^*^	13	300	56	Veterans without mTBI Hx
Donnell et al. (40)	Cohort	DSM-IV	NS	37.8 (2.5)	NS	0	154	3,001	Veterans without mTBI Hx
Donnelly et al. (41)	Cohort	BDI-II	32.1	33.6 (9.6)	1.5	11	130	225	Veterans without mTBI Hx
Drapeau et al. (42)	Cross-sectional	BDI-II	35.8	36.8 (9.25)	0.9 (0.7)	40	20	11	Healthy control
Dretsch et al. (43)	Cross-sectional	ZDS	NS	26.0 (7.0)	NS	6	167	291	Military without mTBI Hx
Epstein et al. (44)	Cross-sectional	HAMD	34.5	35.5 (7.9)	8.9 (7.8)	0	55	27	Healthy control
Gaines et al. (45)	Cross-sectional	BDI-II	24.5	29.5 (6.1)	5.0	0	57	57	Veterans without mTBI Hx
Gardner et al. (46)	Cross-sectional	DASS	32.5	38.3 (4.6)	5.8	0	16	16	Healthy control
Gill et al. (47)	Cross-sectional	QIDS	29.8	31.3 (6.8)	1.5	7	42	22	Healthy control
Graham et al. (48)	Cross-sectional	CES-D	30.8	31.8 (7.6)	4.3 (2.0)	5	41	26	Veterans without mTBI Hx
Guskiewicz et al. (49)	Cohort	Self-report	29.1	53.8 (13.4)	24.7 (13.7)	0	1,513	1,039	Retired sportsplayer peers without mTBI Hx
Himanen et al. (50)	Cohort	BDI-II	29.1	60.1 (10.6)	31.0 (3.6)	33	17	31	Healthy control
Hoge et al. (6)	Cohort	PHQ-15	NS	NS	NS	1	368	1673	Military without mTBI Hx
Hoot et al. (51)	Cohort	PHQ-9	27.1	36.0^*^	8.9^*^	12	376	73	Veterans and service members without mTBI Hx
Iverson and Pogoda (52)	Cohort	CES-D	NS	51.9 (10.1)	NS	100	33	119	Veterans without mTBI Hx
Johansson et al. (53)	Cross-sectional	Clinical interview	40.2	48.5 (1.7)	6.5 (1.7)	72	46	40	Healthy control
Jurick et al. (54)	Cross-sectional	PHQ-9	28.4	35.0 (8.1)	6.6 (4.4)	9	42	28	Veterans without mTBI Hx
Kerr et al. (55)	Cross-sectional	PHQ-9	NS	NS	NS	0	172	32	Former collegiate football peers without mTBI Hx
Kerr et al. (56)	Prospective observational	GHS	NS	63.1 (11.2)	NS	0	679	365	Retired sportsplayer peers without mTBI Hx
Konrad et al. (57)	Cross-sectional	BDI	30.7	36.7 (12.4)	6.0	48	33	33	Healthy control
LaFrance et al. (58)	Cross-sectional	BDI-II	NS	38.9 (12.6)	NS	66	41	51	Psychogenic non-epileptic seizure (no mTBI Hx)
Lee et al. (59)	Cohort	PHQ-15	22.4	27.0 (5.3)	4.6 (1.9)	10	182	2,843	Military without mTBI Hx
Leveille et al. (60)	Cross-sectional	BDI-II	20.1	22.7 (2.4)	2.6 (1.0)	50	22	28	University athlete peers without mTBI Hx
Lippa et al. (61)	Cross-sectional	WHODAS	28.1	31.2 (7.4)	3.1 (2.4)	8	99	156	Veterans without mTBI Hx
Losoi et al. (62)	Prospective longitudinal	BDI-II	36.5	37.0 (11.8)	0.5	39	69	37	Orthopedic injury
Mac Donald et al. (63)	Prospective observational	DSM-IV; MADRS	27.0	32.0 (8.0)	5.0	7	50	44	Military without mTBI Hx
Mac Donald et al. (64)	Prospective observational	BDI	NS	26.0^*^	5.0	8	38	34	Military without mTBI Hx
MacGregor et al. (65)	Cohort	PDHRA	22.6	23.3	0.7	0	334	658	Military without mTBI Hx
Maruta et al. (66)	Cross-sectional	CES-D	33.3	34.9 (14.0)	1.6	52	33	140	Healthy control
Mickeviciene et al. (67)	Prospective observational	VAS	33.7	36.0 (10.5)	2.3	40	131	146	Age and sex matched minor non-head injury (hospital sample)
Moller et al. (68)	Cross-sectional	HADS	34.3	36.0	1.8 (1.6)	50	24	31	Healthy control
Morey et al. (69)	Cross-sectional	BDI	29.9	39.6 (10.8)	9.7 (10.8)	3	30	70	Healthy control + confirmatory controls
Morissette et al. (70)	Cross-sectional	BDI-II	NS	36.9 (9.4)	NS	4	98	115	Veterans without mTBI Hx
Newberg et al. (71)	Cross-sectional	BDI	50.5	51.0 (16)	0.5	44	25	10	Healthy control
Nordhaug et al. (72)	Cohort	HADS	54.7	59.6 (14.7)	4.9 (3.2)	47	294	25,662	Population cohort
Olivera et al. (73)	Cohort	QIDS	NS	30.4 (4.6)	NS	1	70	28	Military without mTBI Hx
Ozen and Fernandes (74)	Cross-sectional	BDI	13.5	19.8 (1.6)	6.3 (4.6)	49	43	44	University peers without mTBI Hx
Palombo et al. (75)	Cohort	DSM-IV	25.3	29.3 (7.6)	4.0 (2.2)	4	50	25	Veterans without mTBI Hx
Peskind et al. (76)	Cross-sectional	PHQ-9	28.5	32.0 (8.5)	3.5 (1.2)	0	12	12	Healthy control
Petrie et al. (77)	Cross-sectional	PHQ-9	27.8	31.6 (9.2)	3.8 (1.5)	0	34	17	Veterans without mTBI Hx
Pineau et al. (78)	Cross-sectional	BDI-II	35.2	36.8 (15.3)	1.7 (1.4)	44	25	25	Healthy control
Pogoda et al. (79)	Cohort	ICD-9	NS	30.6 (8.2)	NS	5	9,998	3,748	Veterans without mTBI Hx
Polusny et al. (80)	Cohort	BDI-II	30.0	31.0 (8.0)	1.0	3	60	748	Military (National Guard soldiers) without mTBI Hx
Raikes et al. (81)	Cohort	BDI-II	23.9	24.9 (8.6)	0.5	62	5	18	Healthy control
Raskin (82)	Cross-sectional	BDI	38.3	40.4 (11.0)	2.1 (0.8)	100	10	10	Healthy control
Rogers et al. (83)	Cross-sectional	DASS	20.2	21.1 (5.6)	1.3 (0.5)	40	10	10	University peers without mTBI Hx
Schiehser et al. (84)	Cross-sectional	BDI-II	26.4	33.1 (6.9)	6.7 (4.5)	13	60	40	Veterans without mTBI Hx
Schoenhuber and Gentilini (85)	Case-control	ZDS	27.3	28.0 (14.0)	0.8	0	35	35	Case-control pairing (friends/family)
Small et al. (86)	Cohort	HAMD	NS	59.0^*^	NS	0	5	5	Healthy control
Spira et al. (87)	Cross-sectional	PHQ-8	22.2	22.7 (2.7)	0.5	0	98	305	Military without mTBI Hx
Sponheim et al. (88)	Cross-sectional	BDI-II	31.0	33.7 (7.7)	2.7 (0.8)	0	9	8	Healthy control
Strigo et al. (89)	Cross-sectional	BDI-II	23.9	28.8 (6.6)	4.9 (1.9)	0	20	24	Veterans without mTBI Hx
Suhr and Gunstad (90)	Cohort	BDI-II	NS	18.5 (0.8)	NS	62	63	50	University peers without mTBI Hx
Tarazi et al. (91)	Cross-sectional	PAI (depression subscale)	36.1	53.4 (10.3)	17.3 (14.8)	0	45	25	Healthy control
Tremblay et al. (92)	Cross-sectional	BDI	24.0	60.9 (7.5)	36.9	0	15	15	Healthy control (former university athlete)
Vanderploeg et al. (93)	Randomized control	DSM-IV	21.8	37.8 (2.5)	16.0	0	254	3214	Veterans without mTBI Hx
Vasterling et al. (94)	Cross-sectional	CES-D	24.6	25.6 (5.0)	1.0	0	87	84	Military without mTBI Hx
Vasterling et al. (95)	Cross-sectional	BDI	23.3	48.3 (9.0)	25	2	68	692	Veterans without mTBI Hx
Verfaellie et al. (96)	Cross-sectional	BDI	27.5	30.7 (8.7)	3.2 (1.9)	4	53	39	Veterans without mTBI Hx
Waljas et al. (97)	Cohort	BDI-II	36.8	37.8 (13.5)	1.1 (0.1)	56	103	36	Healthy control
Walker et al. (98)	Cohort	CES-D	24.3	25.0^*^	0.7	3	176	40	Military without mTBI Hx
Walker et al. (99)	Cohort	CES-D	25.9	27.0^*^	1.1	6	29	58	Military & veterans without mTBI Hx
Waterloo et al. (100)	Cohort	BDI-II	26.4	27.4 (15.9)	1.0	43	7	7	Healthy control
Wilk et al. (101)	Cross-sectional	PHQ-15	NS	NS	NS	9	260	846	Military without mTBI Hx

*BDI, Beck Depression Inventory; CES-D, Center for Epidemiologic Studies Depression Scale; DSM, Diagnostic and Statistical Manual of Mental Disorders; HAMD, Hamilton Depression Rating Scale; Hx, History; ICD, International Classification of Diseases; MADRS, Montgomery-Åsberg Depression Rating Scale; mTBI, mild traumatic brain injury; NS, not stated; NSI, Neurobehavioral Symptom Inventory; PAI, Personality Assessment Inventory; PDHRA, Post-Deployment Health Assessment; PHQ, Patient Health Questionnaire; QIDS, Quick Inventory of Depressive Symptomatology; VAS, Visual Analog Scale; WHODAS, World Health Organization Disability Assessment Schedule; ZDS, Zung Depression Scale*.

* *value represents median, rather than mean*.

**Figure 2:**
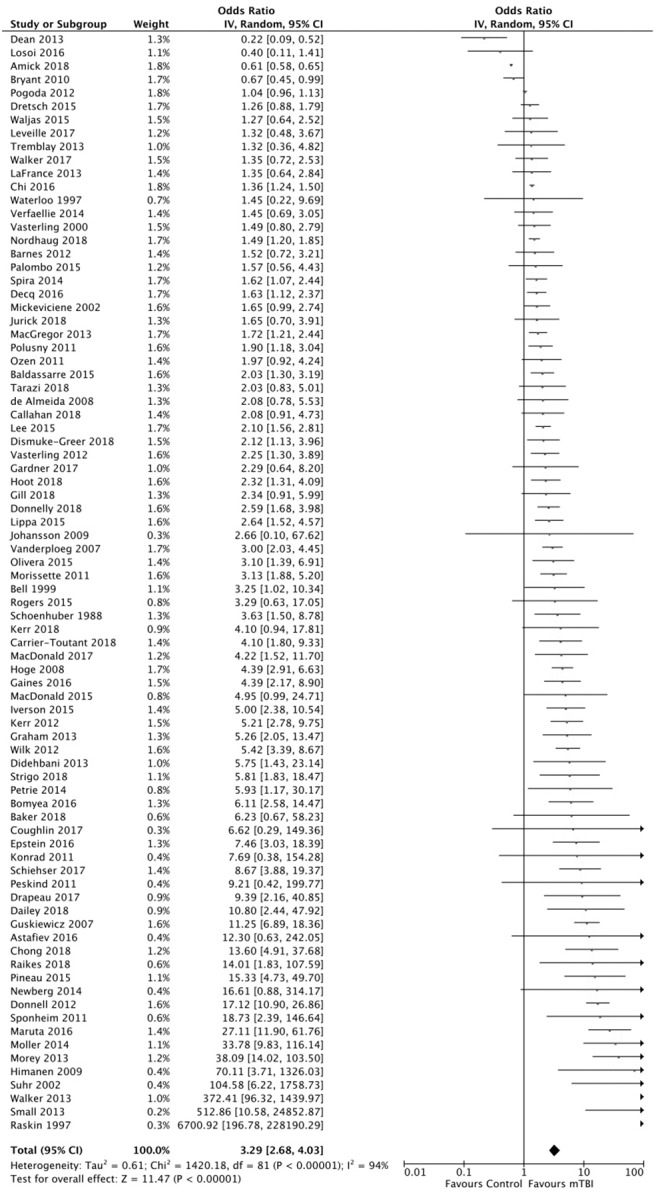
Forest plot representing pooled data from 82 studies reporting depression assessments at least 6 months after mild traumatic brain injuiry (mTBI). In each study, data for mTBI patient groups were compared with control groups for odds of depression. OR, odds ratio. Risk of bias was assessed using the SIGN methodological checklist.

A random-effects meta-analysis was conducted to examine the odds of depression at least 6 months after adult mTBI. We examined Forest plots of continuous and dichotomous data to assess trend similarity (eFigures 1, 2) before pooling these data. The overall OR of depression chronically after mTBI was 3.29 (95% CI 2.68–4.03, *I*^2^ = 96%, *p* < 0.00001) at the meta-analysis level (Figure 2).

Four studies (21, 31, 72, 79) accounted for a large number of the total subjects (365,480/392,834; 93%); however, this contribution largely resulted from control subjects in one study [*n* = 221,501 (31)]. Taking into account the relative frequency of mTBI vs. control subjects, these studies each contributed <2% of the overall weight, for a total contribution of 7.1%. No significant relationship was detected between mTBI and depression when these studies were assessed in isolation (OR 1.06, 95% CI 0.70–1.62; *I*^2^ = 100%, *p* = 0.78). However, removal of these studies from the pooled dataset only resulted in marginal change from the overall OR of post-mTBI depression (OR 3.61, 95% CI 2.89–4.52; *I*^2^ = 85%, *p* < 0.00001).

A number of studies (25, 28, 49, 56, 58, 61, 69, 82, 93–97) described assessment of lifetime depression but did not clearly stipulate whether participants were excluded if screening positively for pre-injury depression. To determine whether these studies unduly influenced findings, we removed these studies and repeated our analysis. We found that the OR remained stable without those studies (OR 3.34, 95% CI 2.96–4.15, *I*^2^ = 94%, *p* < 0.00001) and was similar when these studies were assessed in isolation (OR 3.13, 95% CI 1.72–5.71, *I*^2^ = 94%, *p* = 0.00002).

**Figure 3:**
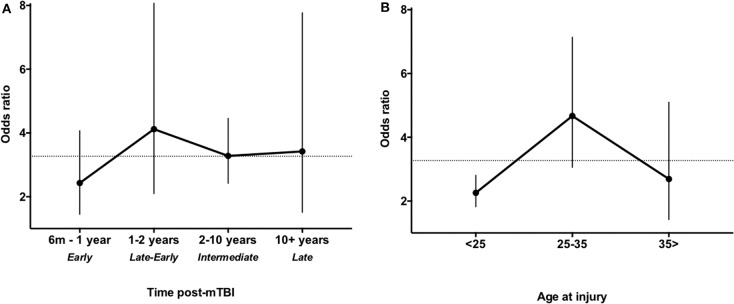
Odds ratio (OR) of depression over time post mild traumatic brain injury (mTBI) **(A)** and age at injury **(B)**. **(A)** Data were stratified into early phase (6 months−1 year post-mTBI), late-early phase (1–2 years post-mTBI), intermediate phase (2–10 years post-mTBI), and late phase (more than 10 years post-mTBI). **(B)** Data were stratified by mean age at injury under 25 years, 25–35 years, or over 35 years. Dashed lines indicate the overall OR.

As a sub-analysis, we investigated the stability of the odds of post-mTBI depression over time. Studies were divided into four categories based on the distribution of time points: 6 months−1 year post-mTBI (nine studies, *n* = 1,906), 1–2 years (17 studies, *n* = 3,526), 2–10 years (27 studies, *n* = 32,093), and more than 10 years post-mTBI (six studies, *n* = 6,339). Twenty-two studies did not report a time interval post-injury and thus were excluded from this sub-analysis. No significant differences were observed between subgroups (*p* = 0.99, one-way ANOVA), although the calculated OR values were uniformly higher following the first subgroup (6 months−1 year). The meta-analysis level ORs and CIs are presented in Figure 3A and were as follows: 6 months−1 year post-mTBI: OR 2.43, 95% CI 1.45–4.07, *I*^2^ = 69%, *p* = 0.0007; 1–2 years: OR 4.12, 95% CI 2.10–8.07, *I*^2^ = 92%, *p* < 0.0001; 2–10 years: OR 3.28, 95% CI 2.42–4.46, *I*^2^ = 76%, *p* < 0.00001; 10+ years: OR 3.23, 95% CI 2.39–4.36, *I*^2^ = 86%, *p* < 0.00001. Three of the included studies performed longitudinal assessment with time points beyond those included in our meta-analysis. Their findings at time points relevant to our study also revealed stable depression symptoms over the following 1 (41), 5 (72), or 9 (56) years post-mTBI, with no apparent reduction in rate of depression. We next investigated whether depression persisted at a similar OR relative to age at mTBI. Studies were divided into those with mean ages of <25 years (18 studies, *n* = 9,622) 25–35 years (29 studies, *n* = 14,652), and more than 35 years of age (12 studies, *n* = 27,243). No significant difference was found between subgroups (*p* = 0.73, one-way ANOVA), although the pooled OR for 25–35 years was numerically higher (Figure 3B). The ORs were as follows: aged under 25: OR 2.26, 95% CI 1.82–2.81, *I*^2^ = 41%, *p* < 0.00001; aged 25–35: OR 4.67, 95% CI 3.06–7.14, *I*^2^ = 87%, *p* < 0.00001; aged over 35: OR 2.69, 95% CI 1.42–5.10, *I*^2^ = 84%, *p* = 0.002.

To examine the possible influence of cohort composition, we further evaluated the impact of age at assessment, biological sex, injury etiology, repeated mTBI, method of depression assessment, and study design. We tested the effect of age at assessment by dividing studies into groups where the mean age was under 40 (63 studies, *n* = 81,860) and over 40 years (15 studies, *n* = 30,650). No significant difference between groups was seen (*p* = 0.25, Student's *T*-test). ORs were as follows: aged under 40: OR 3.14, 95% CI 2.48–3.99, *I*^2^ = 94%, *p* < 0.00001; aged over 40: OR 4.57, 95% CI 2.54–8.24, *I*^2^ = 87%, *p* < 0.00001. Two studies included only females, for which the pooled OR was 147.68, 95% CI 169, 110.38, *I*^2^ = 93%, *p* = 0.16. We also compared data for the four studies which included more than 70% females (*n* = 287) to 55 studies including more than 70% males (*n* = 88,239). No statistical difference was found between males and females (*p* = 0.08, Student's *T*-test). The ORs were as follows: Male: OR 3.00, 95% CI 2.33–3.86, *I*^2^ = 95%, *p* < 0.00001; Female: OR 19.97, 95% CI 2.39–166.93, *I*^2^ = 81%, *p* = 0.006.

The relationship between post-mTBI depression and injury etiology was tested by stratifying studies as sport mTBI (17 studies, *n* = 4,827), military-related mTBI (including blast-related brain injury; 34 studies, *n* = 75,348), MVAs (nine studies, *n* = 30,043), and mTBIs uncategorized or of unspecified etiology (36 studies, *n* = 311,687). There was no statistical difference in the OR of depression relative to any mTBI etiology (*p* = 0.61, one-way ANOVA). The ORs for each category were as follows: sports-related injury: OR 3.51, 95% CI 2.01–6.11, *I*^2^ = 78%, *p* < 0.00001; military injury: OR 3.19, 95% CI 2.36–4.32, *I*^2^ = 96%, *p* < 0.00001; MVA: OR 2.3, 95% CI 1.36–3.88, *I*^2^ = 81%, *p* = 0.002; other injury: OR 3.26, 95% CI 2.45–4.35, *I*^2^ = 88%, *p* < 0.00001.

Repetitive injuries were reported in 35/82 (41%) of studies, with 30 (86%) of these studies reporting a majority of subjects with repetitive mTBIs. The odds of depression after repetitive or single mTBI were identical, with an OR for repetitive injuries of 3.66 (95% CI 2.56–5.22, *I*^2^ = 90%, *p* < 0.00001) and an OR for single injuries of 3.48 (95% CI 2.56–5.73, *I*^2^ = 96%, *p* < 0.00001).

The impact of the most frequently utilized six depression assessment methods on OR of depression after mTBI is presented in eResults 2, with no statistical difference in the OR of depression relative to assessment method. The influence of study design (cohort or cross-sectional) is presented in eResults 3, with no statistical difference observed in the odds of depression between these study designs.

**Figure 4:**
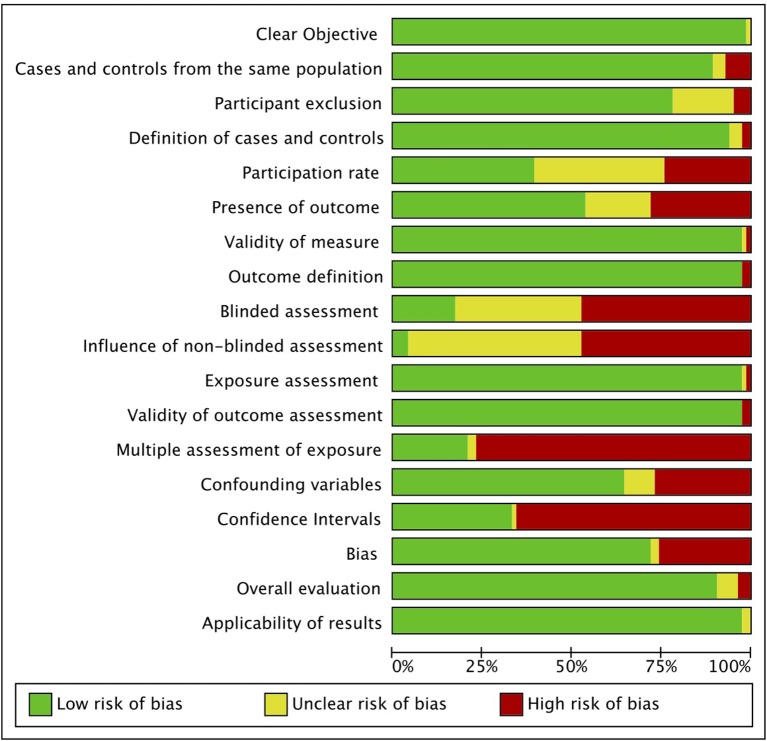
Risk of bias graph. Authors' judgments for each risk of bias item are presented as percentages across all included studies.

Risk of bias is presented in Figure 4 (and eTable 2, in full), with bias risk expressed as a percentage for each assessed item. Risk of bias was greatest for multiple assessment of mTBI exposure (reported in <25% of studies), provision of CIs, and blinding of assessment, which was infrequently reported. The vast majority of studies reported a clear objective, had an appropriate and well-defined control group, and used validated measures of depression and mTBI, with an overall evaluation of low risk of bias and high applicability of results. A funnel plot of the pooled data (eFigure 3) demonstrated substantial asymmetry, representing publication bias. The majority of studies were clustered in the top section of the plot, indicative of more precise effect estimates, with a low number of low-powered studies reporting ORs <1. Orwin's Failsafe-N formula (17) showed that 115 additional studies with an OR of 1.0 would be required to decrease the effect size below a Cohen's *d* of 0.5.

## Discussion

In this meta-analysis of 82 studies and 392,834 participants (96,709 mTBI subjects and 296,125 controls), we found that the OR for depression associated with mTBI (more than 6 months post-injury) was 3.29 when compared with those without a history of mTBI. Given the substantial prevalence of concussion in society, mTBI should now be considered as a significant driver of the overall population risk of depression.

A key secondary finding was that odds of depression occurring after mTBI are maintained or may even increase with chronicity and do not dissipate in the acute or subacute period. This finding indicates that “mild” TBI has repercussions extending over years, if not decades across a lifetime. Depression in the chronic recovery phase has been most frequently reported for moderate or severe injuries (102), likely because the higher degree of clinical management and follow-up these patients require renders them more accessible to researchers. In contrast to TBIs classed as moderate or severe, those experiencing mTBI are typically discharged rapidly with no or only short-term follow-up. The current Centers for Disease Control and Prevention guidelines (103) specify that patients with isolated mTBI and normal neurological evaluation can be discharged if head CT is negative, with no follow-up required unless post-concussive symptoms persist.

The consensus has been that the emotional sequelae of mTBI generally resolve by 12 weeks post-injury (104). Our data provide the first large-scale evidence to contradict this notion, highlighting a need for management strategies for mTBI to be reexamined. Further comprehensive longitudinal studies are required to elucidate this chronology. To date, a single longitudinal study has documented chronological rates of depression after TBI, following subjects (*n* = 423) monthly or every 2 months over a year (105). The authors found that depression was transient acutely but reemerged in the chronic phase, with a prevalence of 31% at 1 month and 21% at 6 months. This supports previous work suggesting that post-mTBI depression is biphasic, with transient symptomology acutely (13, 105) and a reemergence chronically (106). Together with this finding, our data highlight the importance of longitudinal assessment to thoroughly characterize emotional disturbance beyond the acute and subacute recovery phases (107).

We found no significant relationship between depression and the age at which mTBI occurred, although the pooled OR for those aged 25–35 years was numerically higher than those aged under 25. Several studies have now demonstrated a relationship between age at injury and risk of depression (12, 14, 105), whereby experiences of depression increase with advancing age. Although poorly understood, this may be influenced by a decrease in synaptic pruning, reduced neural plasticity, and/or diminished neurogenesis (108) as accompaniments to both depression and older age. Further research is required to determine whether a specific age vulnerability to post-mTBI depression truly exists, with potential need for age-specific management guidelines.

The role of biological sex in mTBI outcome is one of growing interest as females increasingly participate in collision sports and combat roles in military operations. Of 82 studies we included, only two exclusively examined females, while 24 focused only on male subjects. Given the disparity in outcome recoveries (11), more research is clearly needed to identify sex-specific consequences of mTBI. Including studies with more than 70% of either sex, we did not observe a statistical difference in depression, although the pooled OR for females was higher than males. There was substantial disparity between the number of subjects in these studies, potentially increasing the CIs. Ambiguity exists around the susceptibility of female sex to post-TBI depression, with several studies reporting increased post-mTBI depression in females (12, 109), while others have found no such relationship (13).

Blast neurotrauma and militarily acquired injuries are often excluded in meta-analyses on the assumption that these are polytrauma cases with a complicated pathology. We chose to include military injuries in our analysis to provide a full assessment of the scope of depression post-mTBI without imposing any prior assumptions on the data. While the injury modality may differ, many of these forms are also likely to include a polytrauma component (e.g., MVA, assaults) or have injury multipliers, as is the case for collision sports in which the concussed subject lands heavily on the ground. We found that the OR for depression in the chronic recovery phase was comparable no matter the etiology, suggesting that the mode of injury is not a significant factor in post-mTBI depression. This finding echoes two recent studies comparing blast vs. non-blast TBI, which reported similarities in the rate of post-traumatic outcomes (61, 110).

We also found that, at present, there is no compelling evidence for a cumulative effect of mTBI on post-mTBI depression, though further research is certainly required. Repetitive mTBI was documented almost entirely in the sports or military mTBI categories, for which subjects may experience many mTBIs across a career. The influence of repetitive injuries on emotional outcomes is one of contention, with several publications [including a recent systematic review of 47 studies (111)] finding that repetitive mTBI is associated with deteriorating mental health (56, 87), while others have found little psychological consequences of repeated injuries (112).

Our findings should be considered in the context of several important limitations. First, bias in subject selection may have been present in these longer-term studies either by direct selection of patients for which post-concussive symptoms predominates or selection bias due to lingering problems. We found that studies overwhelmingly focused on young adults and the middle aged rather than older participants, and thus our findings may not extend to post-mTBI depression in elderly participants who are more likely to sustain mTBI from other causes, e.g., falls. We also chose not to include pediatric populations, which would require a separate analysis to examine post-mTBI depression in the context of neurodevelopment. Participants in included studies may also have experienced concomitant disorders such as anxiety or posttraumatic stress disorder (PTSD), which might influence results toward an increase or decrease in risk (11). A key inclusion criterion for our research was the presence of a validated instrument to assess the likely presence or absence of depression, chosen to ensure a maximal synthesis of the available data. Aside from structured interviews, a general limitation of these instruments is their lack of confirmatory diagnoses. However, symptom-based scales are frequently used as screening tools for depression and are unlikely to give false negatives. We also did not specify a particular severity of depression in order to avoid bias introduced by any possible threshold effect. We excluded studies for which lifetime or pre-injury depression status was documented; however, the presence of pre-injury depression could not be ruled out for the majority of studies, and therefore we cannot be certain that depression only emerged after injury. To answer this, future studies would need to employ a longitudinal design in a non-injured population (e.g., sportspeople or military) with thorough lifetime assessment of depression and monitor emergence of depression over months and years post-injury.

Our inclusion of studies with a mean time post-injury of 6 months meant that few studies had a lower time limit of <6 months. The majority of studies we included were non-randomized cohort, case-control, cross-sectional, and prospective observational studies, and the inherent heterogeneity and risk of bias for such studies (as compared to randomized controlled trials) should be weighed in context of our findings. While the risk of bias overall was low, we found significant heterogeneity and publication bias, with the majority of high-powered studies showing either a positive or negative effect coupled with small standard errors. A small number of studies were low powered with positive findings; there were no low-powered negative studies that met our inclusion criteria. This is likely a consequence of “file-drawer” bias, for which smaller studies with negative findings are less likely to be published. Using Orwin's Failsafe-N formula, we found that an additional 115 low-powered negative studies would be required to skew the data toward the null hypothesis, providing confidence that our findings are robust. The high degree of between-study heterogeneity could not be explained by subgroup analyses and could potentially limit generalizability of the study results.

Our data show a positive relationship between mTBI and depression in the chronic phase of injury recovery. The size of this effect is substantial, representing a >3-fold increase in risk of depression after 6 months post-mTBI. This finding should prompt a reevaluation of our standard of care for 42 million mTBI cases occurring per year worldwide.

## Author Contributions

SH, CB, and SG conceived the study. SH and CB performed literature search. SH, CB, and TW reviewed literature for suitability to study criteria and collected data. SH, CB, TW, and SG analyzed the data, interpreted the data, and critically reviewed the manuscript. SH made figures. SH, TW, and SG wrote the manuscript.

## Conflict of Interest

The authors declare that the research was conducted in the absence of any commercial or financial relationships that could be construed as a potential conflict of interest.
