# Supplementary material for: Characterizing the Risk of Depression Following Mild Traumatic Brain Injury: A Meta-Analysis of the Literature Comparing Chronic mTBI to Non-mTBI Populations
Source: Front Neurol. 2020 May 19;11:350. doi: 10.3389/fneur.2020.00350 (PMC7248359; doi:10.3389/fneur.2020.00350)
Supplement: Supplementary file 1 [file Data_Sheet_1.docx]

Supplementary Material

Sarah C Hellewell, Caerwen S Beaton, Thomas Welton, Stuart M Grieve. **What is the frequency of depression in the chronic phase of mild traumatic brain injury? A meta-analysis of the literature**

**eMethods. Search strategy; selection criteria – further details; data analysis – further details.**

**eFigure 1. Forest plot of studies using continuous data to report an incidence of depression at least 6 months after mTBI.**

**eFigure 2. Forest plot of studies using dichotomous data to report an incidence of depression at least 6 months after mTBI.**

**eFigure 3. Funnel plot of 82 studies examining depression in the chronic phase of mTBI recovery.**

**eResults 1. Influence of control comparison group makeup on odds ratio of depression after mTBI.**

**eResults 2. Influence of depression assessment method on odds ratio of depression after mTBI.**

**eResults 3. Influence of cross-sectional and cohort study design on odds ratio of depression after mTBI.**

**eTable 1. MOOSE Checklist for Meta-analyses of Observational Studies.**

**eTable 2. Full results of risk of bias assessment.**

**eMethods - search strategy**

**PubMed (MEDLINE)**

1. Mild traumatic brain injury
2. mTBI
3. Brain concussion[MeSH Major Topic]
4. Brain injuries[MeSH Major Topic]
5. Craniocerebral Trauma[MeSH Major Topic]
6. Depression[MeSH Major Topic]
7. Depressive disorder
8. Major depressive disorder
9. MDD
10. Mood disorder

**Search (#1 OR #2 OR #3 OR #4 OR #5) AND (#6 OR #7 OR #8 OR #9 OR #10)**

**Filters: Human, English**

**Web of Science**

TOPIC: (depression OR "depressive disorder" OR "major depressive disorder") AND TOPIC: ("brain concussion" OR "mild traumatic brain injury" OR mTBI OR Craniocerebral Trauma OR concussion)

**Filters: Human, English**

**ProQuest**

TOPIC: (depression OR "depressive disorder" OR "major depressive disorder") AND ("brain concussion" OR "mild traumatic brain injury" OR mTBI OR “Craniocerebral Trauma” OR concussion)

**Filters: Human, Adult, English**

**eMethods - study selection criteria – further details**

Narrative review articles, books, non-peer or limited-review conference proceedings, conference abstracts, dissertations and news articles were excluded. Full text articles and their citations were downloaded and exported to Endnote (X7, Thompson Reuters, Ontario, Canada).

Studies were excluded if they did not meet the following inclusion criteria: adult human subjects, at least one instance of mTBI, chronic time-point (more than six months post-injury), and validated measure of depression in accordance with the standard operationalised diagnostic criteria (Feighner criteria, Research Diagnostic Criteria, DSM-III, DSM-III-R, DSM-IV, DSM-5, and ICD-10). Studies were also excluded if authors described presence of lifetime of pre-injury depression.

Due to a lack of standardized mTBI diagnostic criteria, mTBI was assessed in myriad ways. The most frequent criteria applied were the American Congress of Rehabilitation Medicine (ACRM) Mild Traumatic Brain Injury Committee Criteria, defined by:

Any period of loss of consciousness (LOC); any loss of memory for events immediately before or after the accident; any alteration in mental state (AMS) at the time of the accident; and any focal neurological deficit(s) that may or may not be transient; but where severity does not exceed loss of consciousness of 30 minutes or less; Glasgow Coma Scale (GCS) of 13-15 after 30 minutes; post-traumatic amnesia not greater than 24 hours.

Studies not using ACRM criteria typically assessed the same factors with more or less stringent criteria, e.g. LOC <20 min or present in an undefined time; PTA of any duration, GCS 14-15. All studies were included as long as sufficient evidence was provided to satisfy the requirement of mTBI.

Studies straddling exclusion criteria were discussed with a necessitation of agreement between at least two authors. Studies for discussion were those reporting mild and moderate TBI together, where the majority of the population met a mild criteria; studies for which the lower age limit was below 18 but the mean was above 18; studies for which the lower limit of time post-injury was below six months but the mean was above six months; and studies not specifying a post-injury time but for which an approximate time could potentially be extrapolated (e.g. in the case of subjects injured during military operations for which theatre dates were known).

After full screening, reference lists of included studies were searched for any results that were not returned by our database search.

**eMethods – data analysis – further details**

In the case of continuous data reported as mean and 95% confidence interval, the standard deviation of the dataset was manually calculated. Military studies variably classified injuries, with the most common convention being ‘blast’ or ‘non-blast’ injury types. Since a significant proportion of studies combined analysis for blast and non-blast, these studies were classified under the all-inclusive general aetiology of ‘military injuries’. Studies for which no injury modality was specified (or for which no category existed) were classed as ‘other/unclassified’ injuries. This category also included falls, workplace injuries, assaults, and domestic violence injuries.

Odds ratios were determined using The Cochrane Collaboration RevMan software, with the odds ratio calculated as the ratio of the probability of depression being present compared to the probability of it being absent, where the odds of depression in mTBI is divided by the odds of depression in controls.

**eFigure 1. Forest plot of studies using continuous data to report an incidence of depression at least 6 months after mTBI.** In each study, data for mTBI patient groups were compared with control groups for rates of depression.

**eFigure 2. Forest plot of studies using dichotomous data to report an incidence of depression at least 6 months after mTBI.** In each study, data for mTBI patient groups were compared with control groups for rates of depression.

**eFigure 3. Funnel plot of 82 studies examining depression in the chronic phase of mTBI recovery.** Dashed line indicates pooled OR. OR = odds ratio, SE = standard error.

**eResults 1. Influence of control comparison group makeup on odds ratio of depression after mTBI.**

Our work assessed studies comprising control cohorts from variable sources. In order to determine whether control cohort makeup influenced our findings, we performed post-hoc examination of studies comparing non-mTBI injury or illness, and healthy control or population sample.

There were five non-mTBI injury or illness control studies for which, the OR of depression chronically after mTBI was 1.14 (95% CI, 0.63, 2.06; *I^2^*=72%, p = 0.67). This group could be further divided into three who recruited injured (non-mTBI) controls (e.g., othopaedic injuries) and two who examined non-mTBI neurological illness controls (migraineurs and psychogenic nonepileptic seizure). For the non-mTBI injury controls, the OR of depression chronically after mTBI was 0.85 (95% CI, 0.40, 1.82; *I^2^*=78%, p = 0.68). For the non-mTBI neurological illness controls, the OR of depression chronically after mTBI was 1.87 (95% CI, 0.81, 4.29; *I^2^*=36%, p = 0.14).

**eResults 2. Influence of depression assessment method on odds ratio of depression after mTBI.**

Our study inclusion criteria stipulated that a validated measure of depression was used in accordance with standard operationalized diagnostic criteria (see eMethods). This meant that data were drawn from studies utilising both diagnostic interview and self-report questionnaires. In order to examine whether measures were more or less likely to assign depressive status, we performed post-hoc examination of the most frequent 6 assessment methods separately.

Beck Depression Inventory (BDI/BDI-II) – 35 studies: The OR of depression chronically after mTBI as assessed using the BDI or BDI-II was 3.98 (95% CI, 2.85, 5.56; *I^2^*=76%, p < 0.00001).

Patient Health Questionnaire (PHQ) – 11 studies: The OR of depression chronically after mTBI as assessed using the PHQ was 2.68 (95% CI, 1.84, 3.9; *I^2^*=70%, p < 0.00001).

Centre for Epidemiologic Studies Depression Scale (CES-D) – 7 studies: The OR of depression chronically after mTBI as assessed using the CES-D was 9.07 (95% CI, 2.76, 29.89; *I^2^*=93%, p = 0.0003).

Diagnostic and Statistical Manual of Mental Disorders (DSM-IV) clinical interview – 4 studies: The OR of depression chronically after mTBI as assessed using the DSM-IV clinical interview was 2.95 (95% CI, 0.87, 10.02; *I^2^*=96%, p = 0.08).

Hospital Anxiety and Depression Scale (HADS) – 4 studies: The OR of depression chronically after mTBI as assessed using the HADS was 2.04 (95% CI, 0.49, 8.52; *I^2^*=93%, p = 0.33).

Hamilton Depression Rating Scale (HAMD) clinical interview – 3 studies: The OR of depression chronically after mTBI as assessed using the HAMD clinical interview was 16.92 (95% CI, 1.99, 143.61; *I^2^*=54%, p = 0.01).

Note: All other depression measures were assessed in a maximum of two studies, and were thus omitted from this analysis. **eResults 3. Influence of cross-sectional and cohort study design on odds ratio of depression after mTBI.**

Our work assessed studies with variable recruitment methods, with the majority designed as either cross-sectional or cohort studies. In order to determine whether recruitment method and study design influenced results, we performed post-hoc examination of cohort and cross-sectional studies separately.

For the 48 cross-sectional studies, The OR of depression chronically after mTBI was 3.74 (95% CI, 2.45, 5.45; *I^2^=*93%, p < 0.00001).

For the 26 cohort studies, The OR of depression chronically after mTBI was 3.13 (95% CI, 2.32, 4.22; *I^2^=*94%, p < 0.00001).

Given that these odds ratios and heterogeneity are similar, these results suggest that heterogeneity likely arises from other methodological issues or sampling biases as discussed in the discussion section, and is not due to recruitment method.

**
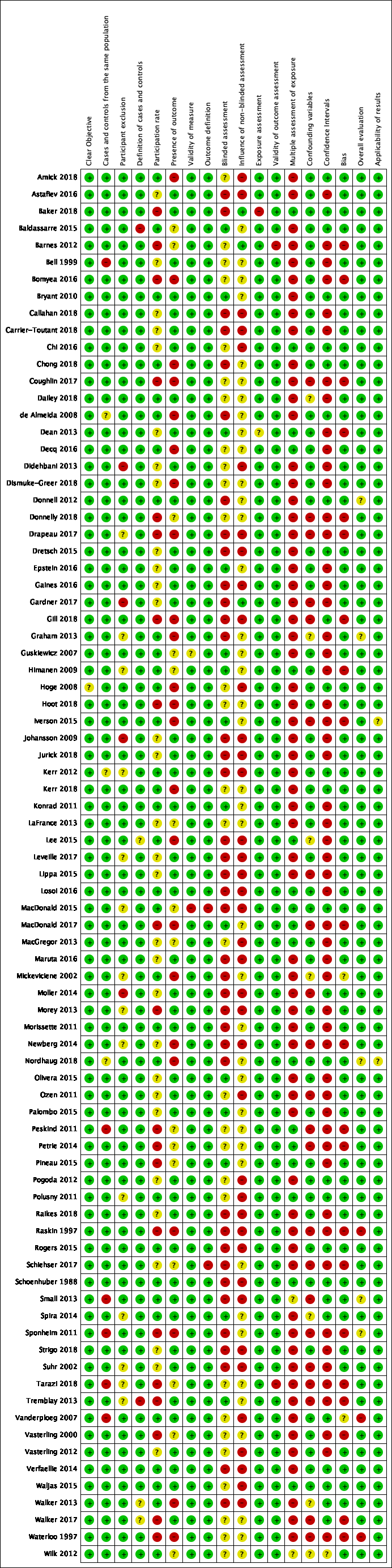
eTable 2. Full results of risk of bias assessment.**

Green plus sign = met criteria; yellow question mark sign = not enough information provided to make a decision on criteria; red minus sign = did not meet criteria.

**eTable 1. MOOSE Checklist for Meta-analyses of Observational Studies.**

| **Item No** | **Recommendation** | **Reported on Page No** |
| --- | --- | --- |
| Reporting of background should include | | |
| 1 | Problem definition | 4 |
| 2 | Hypothesis statement | - |
| 3 | Description of study outcome(s) | 5 |
| 4 | Type of exposure or intervention used | 6, Supplement |
| 5 | Type of study designs used | 5 |
| 6 | Study population | 4,6 |
| Reporting of search strategy should include | | |
| 7 | Qualifications of searchers (eg, librarians and investigators) | Title page |
| 8 | Search strategy, including time period included in the synthesis and key words | 4,5, Supplement |
| 9 | Effort to include all available studies, including contact with authors | 5 |
| 10 | Databases and registries searched | 5, Supplement |
| 11 | Search software used, name and version, including special features used (eg, explosion) | 5, Supplement |
| 12 | Use of hand searching (eg, reference lists of obtained articles) | 5, Supplement |
| 13 | List of citations located and those excluded, including justification | 6,7, Table 1 |
| 14 | Method of addressing articles published in languages other than English | - |
| 15 | Method of handling abstracts and unpublished studies | 5, Supplement |
| 16 | Description of any contact with authors | - |
| Reporting of methods should include | | |
| 17 | Description of relevance or appropriateness of studies assembled for assessing the hypothesis to be tested | 6-8 |
| 18 | Rationale for the selection and coding of data (eg, sound clinical principles or convenience) | 6-8, Supplement |
| 19 | Documentation of how data were classified and coded (eg, multiple raters, blinding and interrater reliability) | 6-8, Supplement |
| 20 | Assessment of confounding (eg, comparability of cases and controls in studies where appropriate) | 8, Figure 4, Supplement |
| 21 | Assessment of study quality, including blinding of quality assessors, stratification or regression on possible predictors of study results | 8, Figure 4, Supplement |
| 22 | Assessment of heterogeneity | 6-8, Figure 4, Supplement |
| 23 | Description of statistical methods (eg, complete description of fixed or random effects models, justification of whether the chosen models account for predictors of study results, dose-response models, or cumulative meta-analysis) in sufficient detail to be replicated | 6-8 |
| 24 | Provision of appropriate tables and graphics | Table 1, Figure 2 & 3, Supplement |
| Reporting of results should include | | |
| 25 | Graphic summarizing individual study estimates and overall estimate | Figure 2, Supplement |
| 26 | Table giving descriptive information for each study included | Table 1 |
| 27 | Results of sensitivity testing (eg, subgroup analysis) | 7, 8, Figure 2 & 3 |
| 28 | Indication of statistical uncertainty of findings | 6-8, Supplement |
